# Supplementary material for: An entanglement association polymer electrolyte for Li-metal batteries
Source: Nat Commun. 2024 Mar 20;15:2500. doi: 10.1038/s41467-024-46883-8 (PMC10954637; doi:10.1038/s41467-024-46883-8)
Supplement: Supplementary file 1 — Supplementary Information [file 41467_2024_46883_MOESM1_ESM.pdf]

Supporting information

An entanglement association polymer electrolyte for Li-metal batteries

Hangchao Wang,[a] Yali Yang, [a] Chuan Gao, [a] Tao Chen, [a] Jin Song, [a] Yuxuan Zuo, [a] Qiu Fang, [b] Tonghuan Yang, [a] Wukun Xiao,[a] Kun Zhang, [a] Xuefeng Wang*[bc] and Dingguo Xia* [a,b]

[a.] Beijing Key Laboratory of Theory and Technology for Advanced Batteries Materials, School of Materials Science and Engineering, Peking University, Beijing 100871, PR China.

[b.] Institute of carbon neutrality, Peking University, Beijing 100871, PR China.

[c.] Laboratory for Advanced Materials & Electron Microscopy, Institute of Physics, Chinese Academy of Sciences, Beijing 100190, China; College of Materials Science and Opto-Electronic Technology, University of Chinese Academy of Sciences, Beijing 100049, China

Corresponding authors.

*E-mail: dgxia@pku.edu.cn; wxf@iphy.ac.cn.

Supplemental Figures and Tables

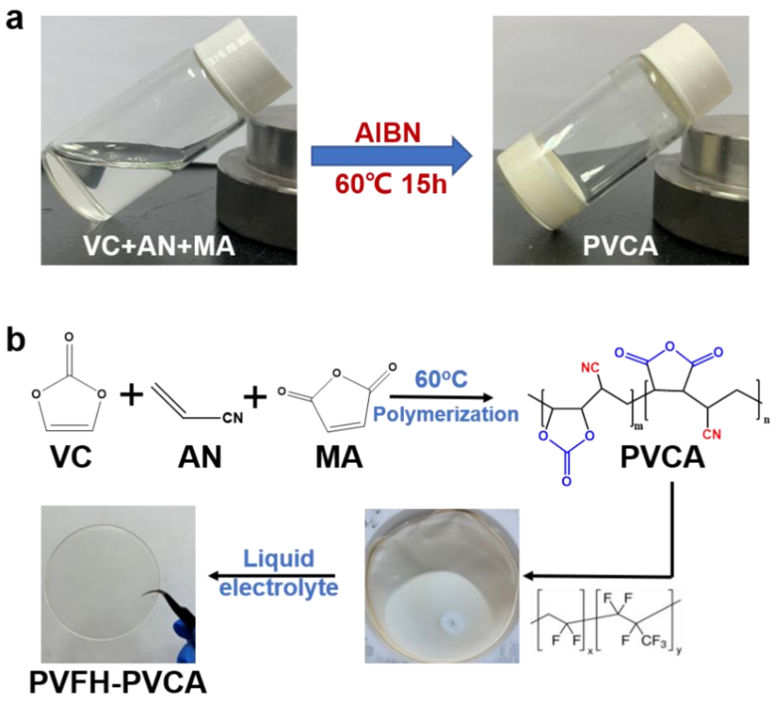

Supplementary Fig. 1. (a) The digital image of polymerization of the VC, AN and MA to form a PVCA polymer after heating at 60°C for 15 h. **(b)** Schematic illustration of the synthesis of the PVFH-PVCA electrolyte. After soaking in liquid electrolyte (1.0M $LiPF_6$,0.02M LiDFOB in FEC:HFE:FEMC=2:2:6 Vol%;), the PVFH-PVCA becomes semitransparent, indicating the formation of quasi-solid polymer electrolytes.

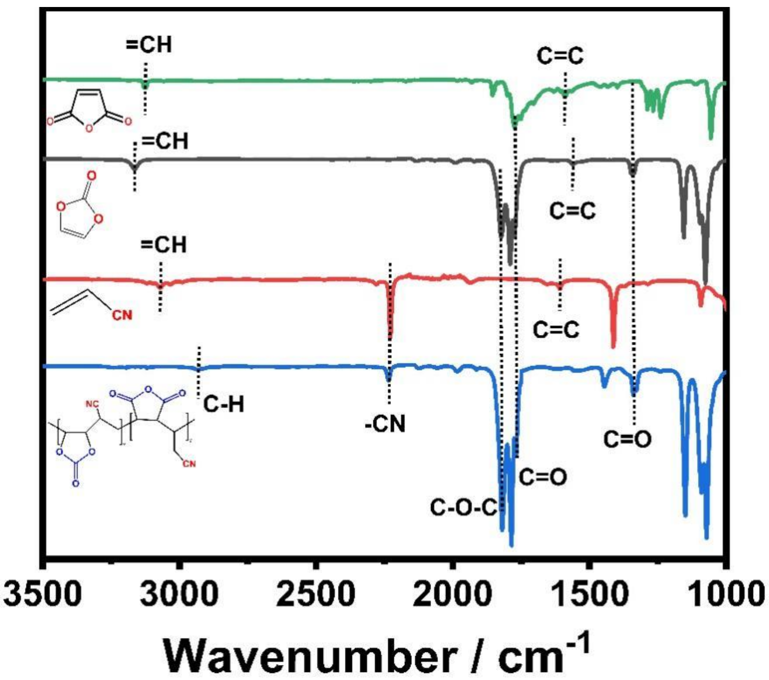

Supplementary Fig. 2. FTIR spectra of the Maleic anhydride (MA), Vinylene Carbonate (VC), Acrylonitrile (AN) and the polymer matrix of PVCA.

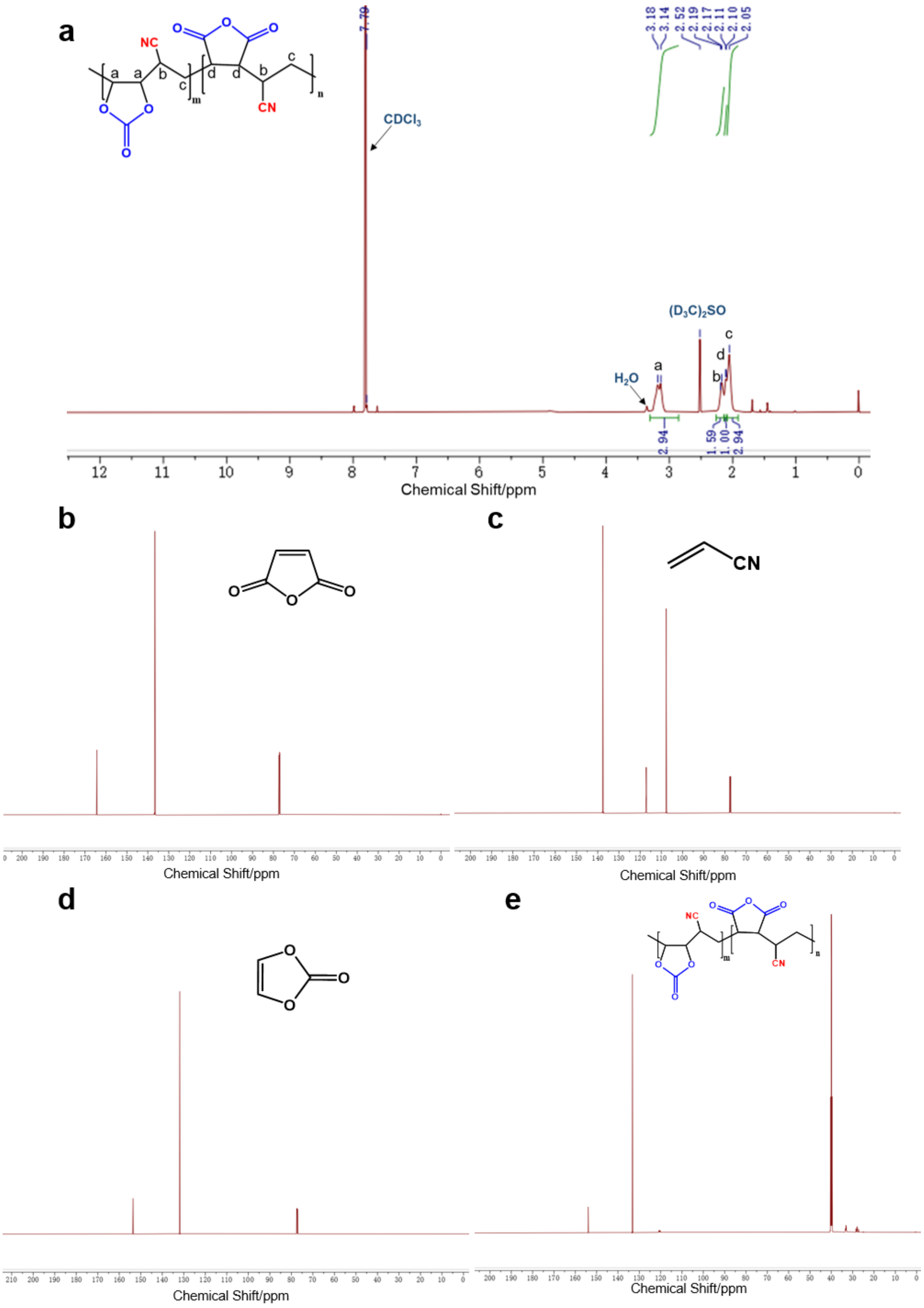

Supplementary Fig. 3. (a) ^{1}H NMR spectrum of the PVCA. ^{13}C-NMR spectrum of the MA **(b).** ^{13}C-NMR spectrum of AN **(c)**. ^{13}C-NMR spectrum of VC **(d)**. ^{13}C-NMR spectrum of PVCA **(e)**.

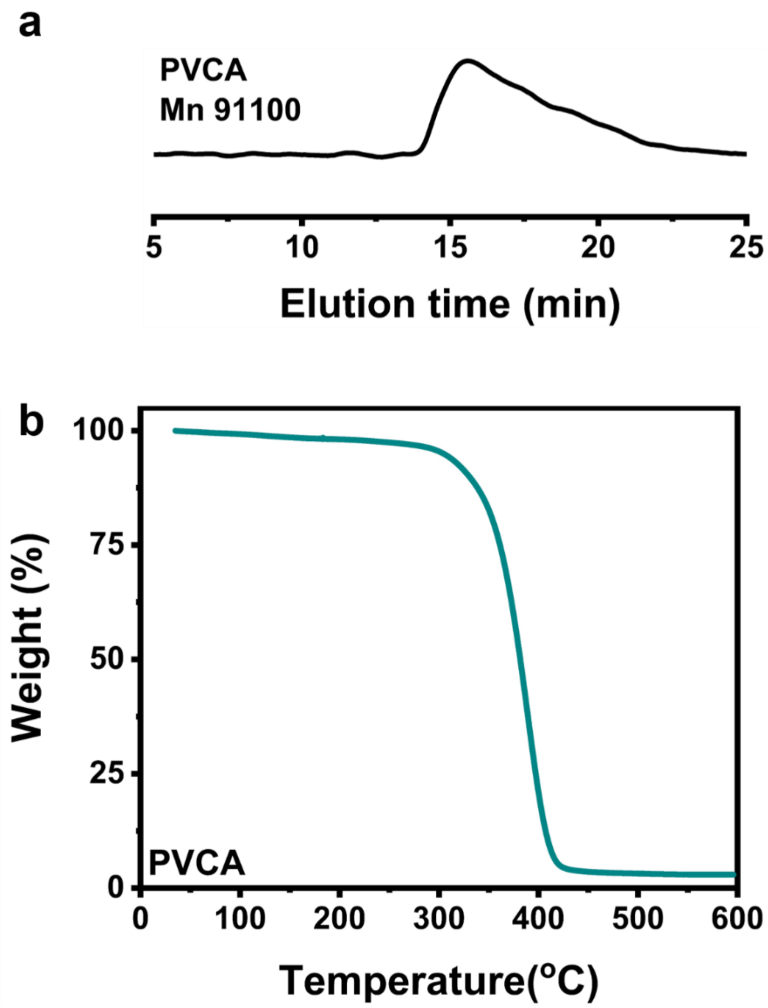

Supplementary Fig. 4. (a) GPC curves of PVCA. **(b)** Thermogravimetric curves of the PVCA.

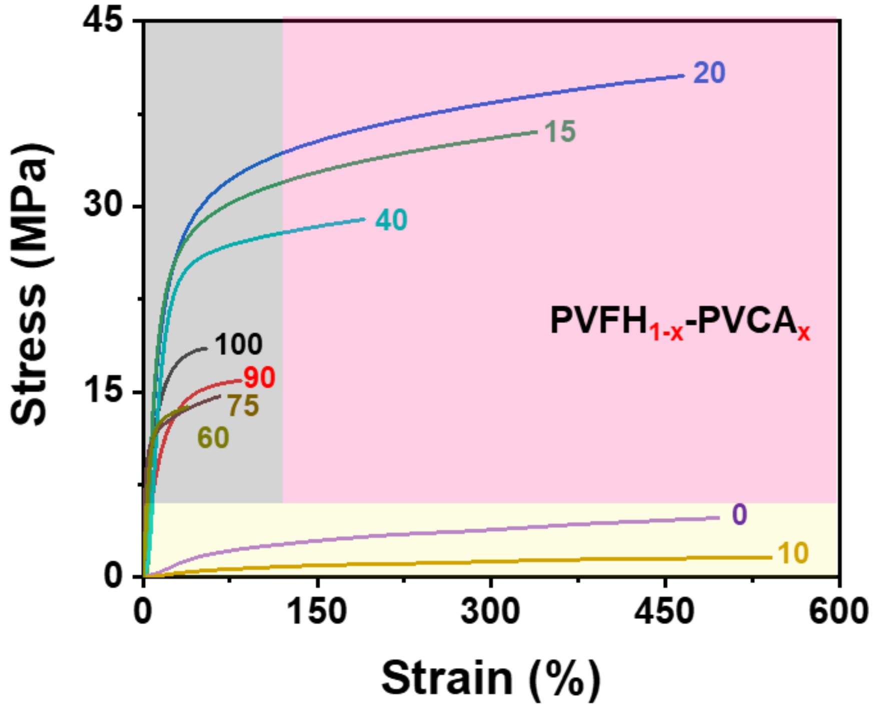

Supplementary Fig. 5. Dynamic mechanical analysis of PVFH-PVCA without liquid electrolyte; X=0~100%.

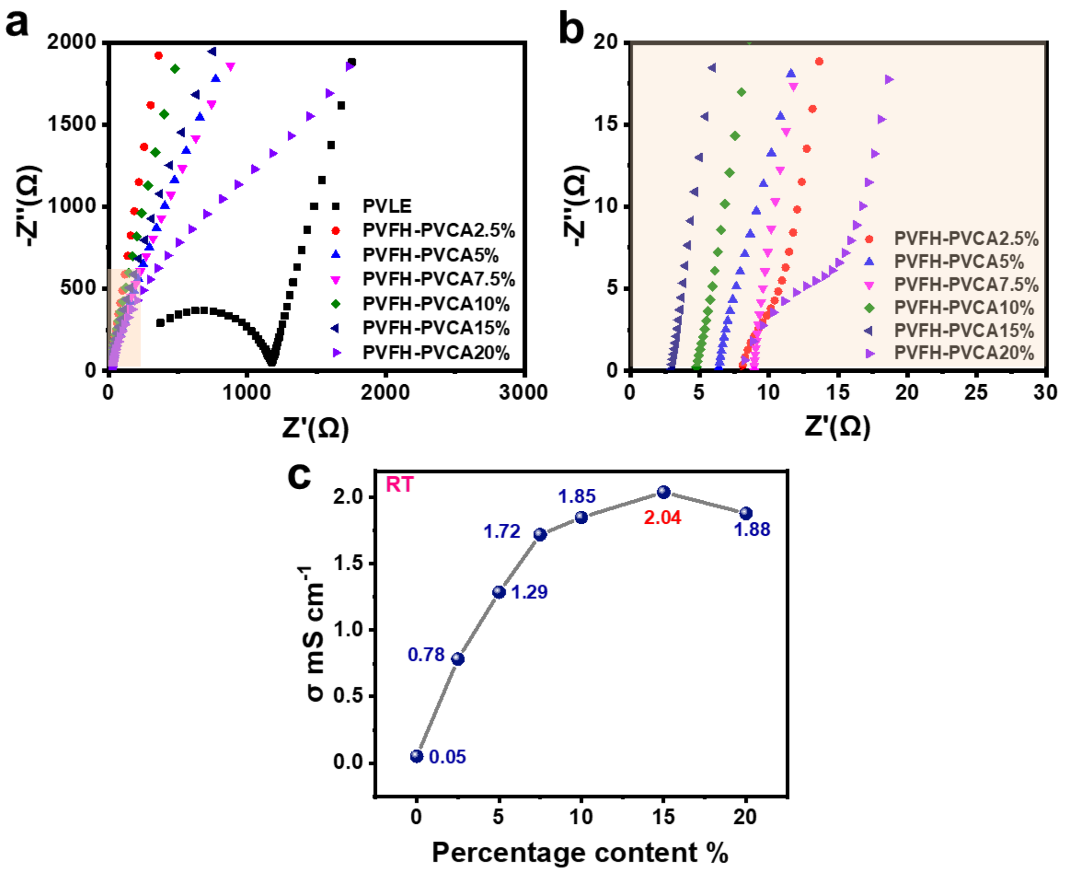

Supplementary Fig. 6. EIS of SS/electrolytes/SS batteries at different content of PVCA (**a**). Enlargement of the shaded area in a that is shown in (**b**) is in the range of 0–30 Ω. (**c**) Room temperature ionic conductivity of PVFH-PVCA with different contents of PVCA.

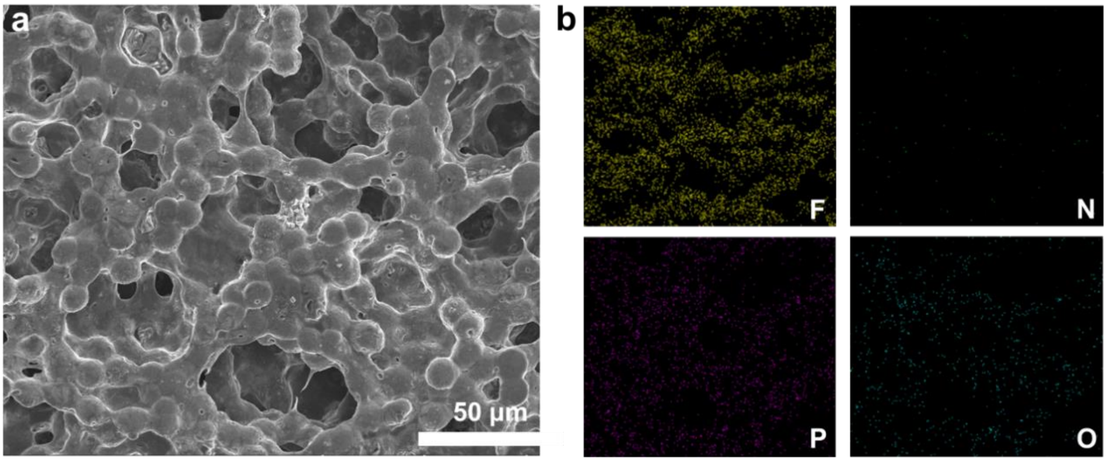

Supplementary Fig. 7. SEM images of surface (**a**) of the PVLE. (**b**) Energy dispersive spectroscopy elemental mappings of F, N, P and O in the PVLE.

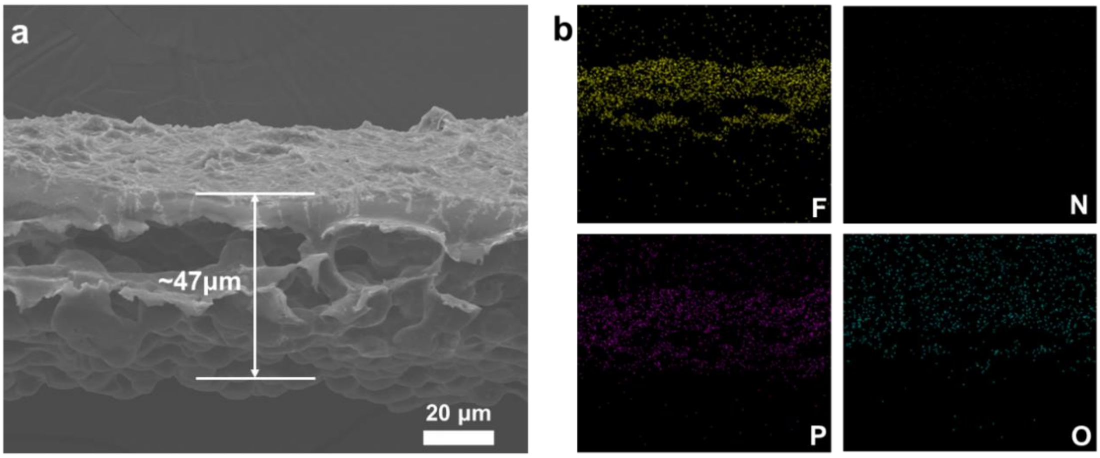

Supplementary Fig. 8. SEM images of the cross-section (**a**) of the PVLE. (**b**) Energy dispersive spectroscopy elemental mappings of F, N, P and O in the PVLE.

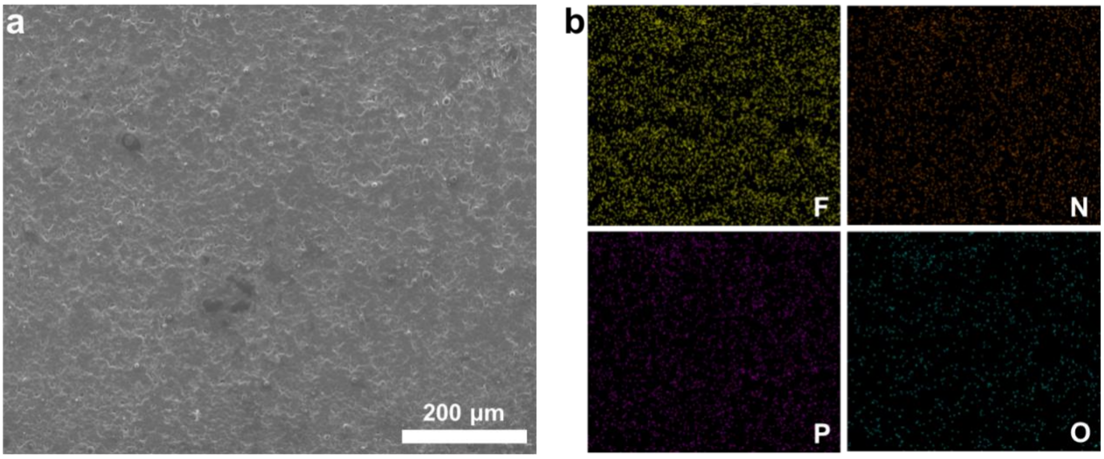

Supplementary Fig. 9.SEM images of surface (**a**) of the PVFH-PVCA. (**b**) Energy dispersive spectroscopy elemental mappings of F, N, P and O in the PVFH-PVCA.

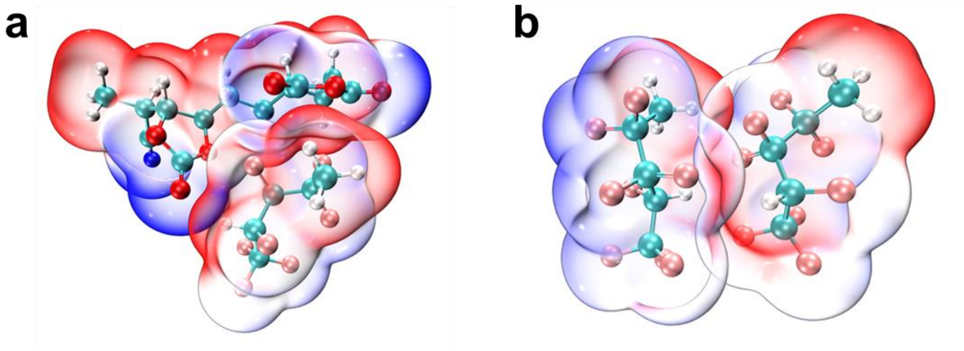

Supplementary Fig.10. Adsorption energy of (a) PVFH with PVCA (-44.461 kJ/mol) and (b) PVFH with PVFH (-12.313kJ/mol).

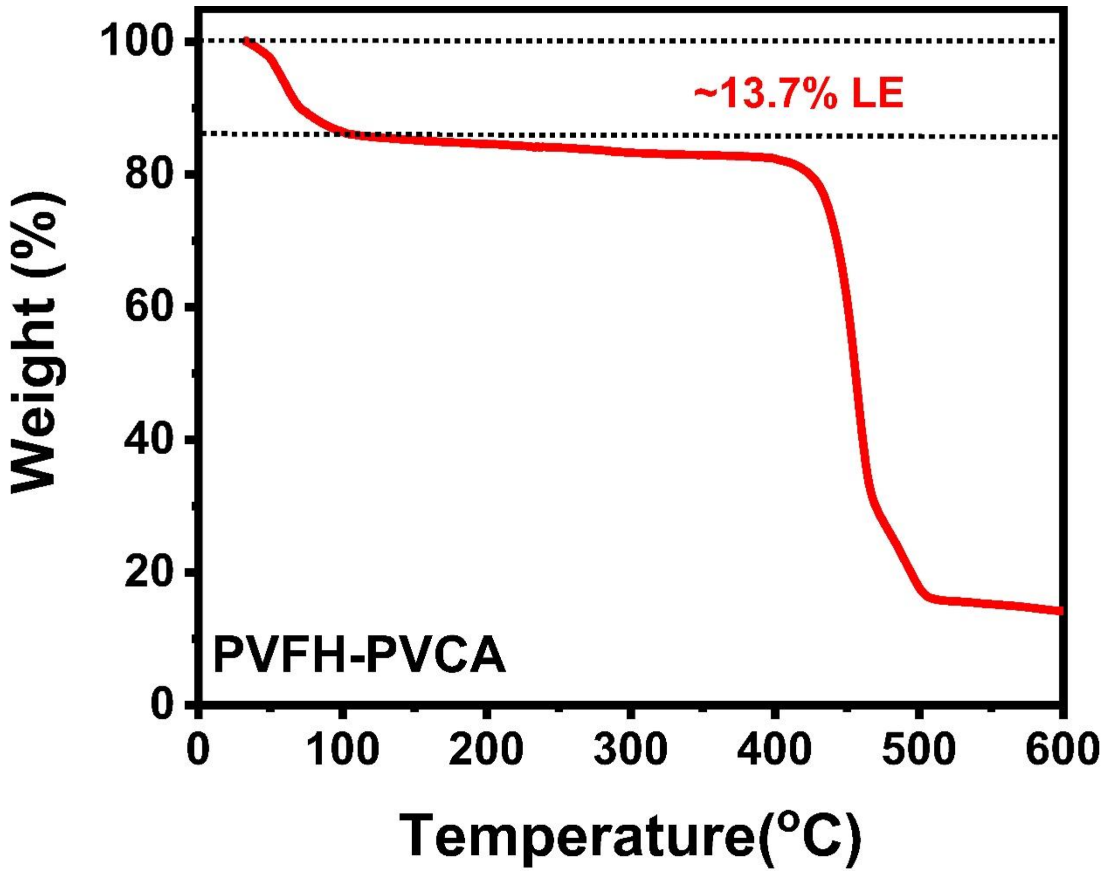

Supplementary Fig. 11. TGA curves of PVFH-PVCA electrolytes at heating rate of 5 ºC min^{-1}.

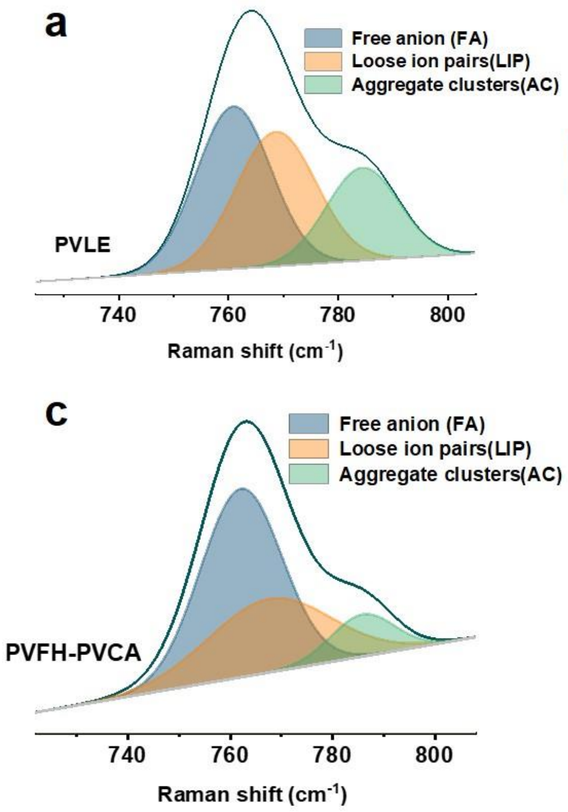

Supplementary Fig. 12. Raman spectra of the PVLE (**a**) and PVFH-PVCA (**c**) electrolytes and corresponding quantification results of the PF_6^- anion states in the electrolytes (**b** and **d**).

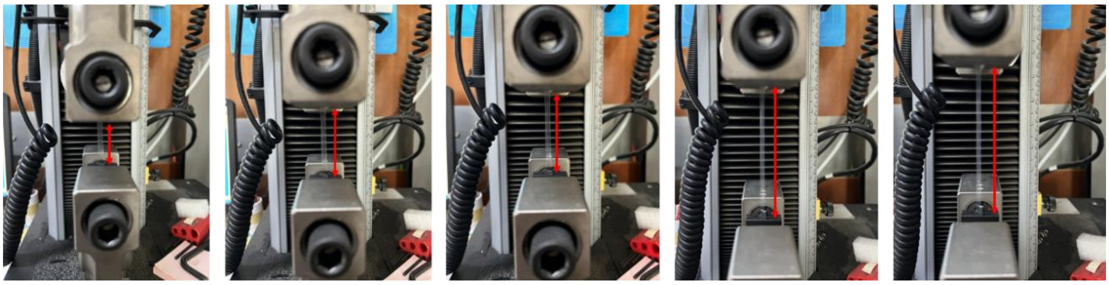

Supplementary Fig. 13. Stress–strain measurement of the PVFH-PVCA membrane.

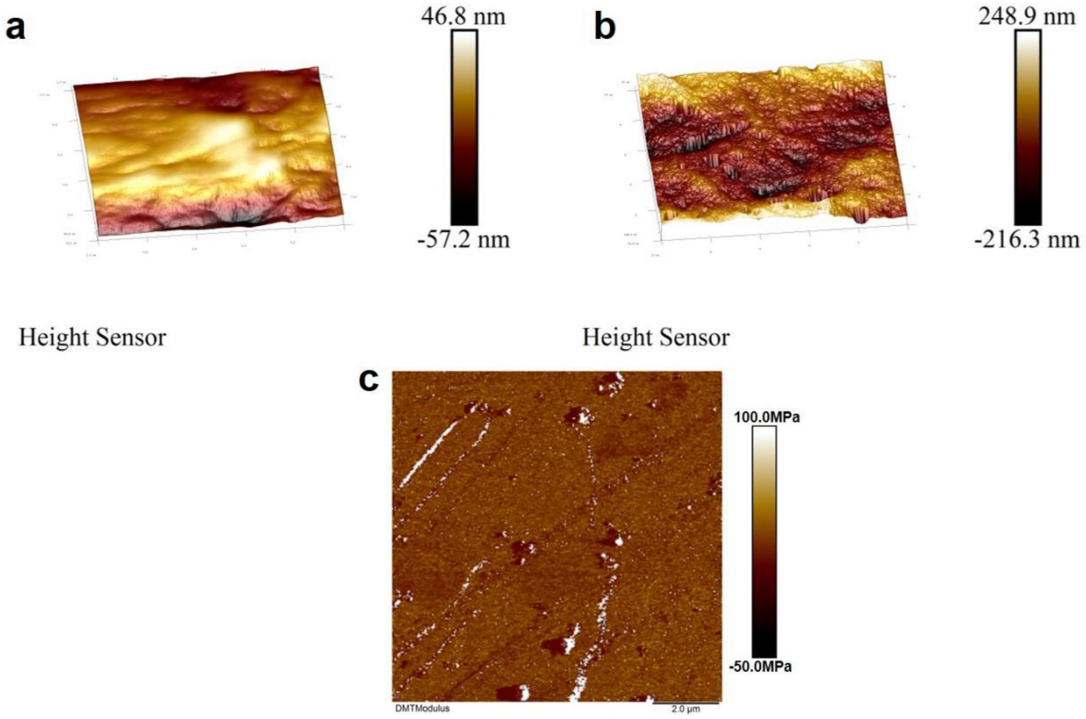

Supplementary Fig. 14. (**a**) AFM images of the PVFH-PVCA and PVLE (**b**) surface. (**c**) Young's modulus of PVLE.

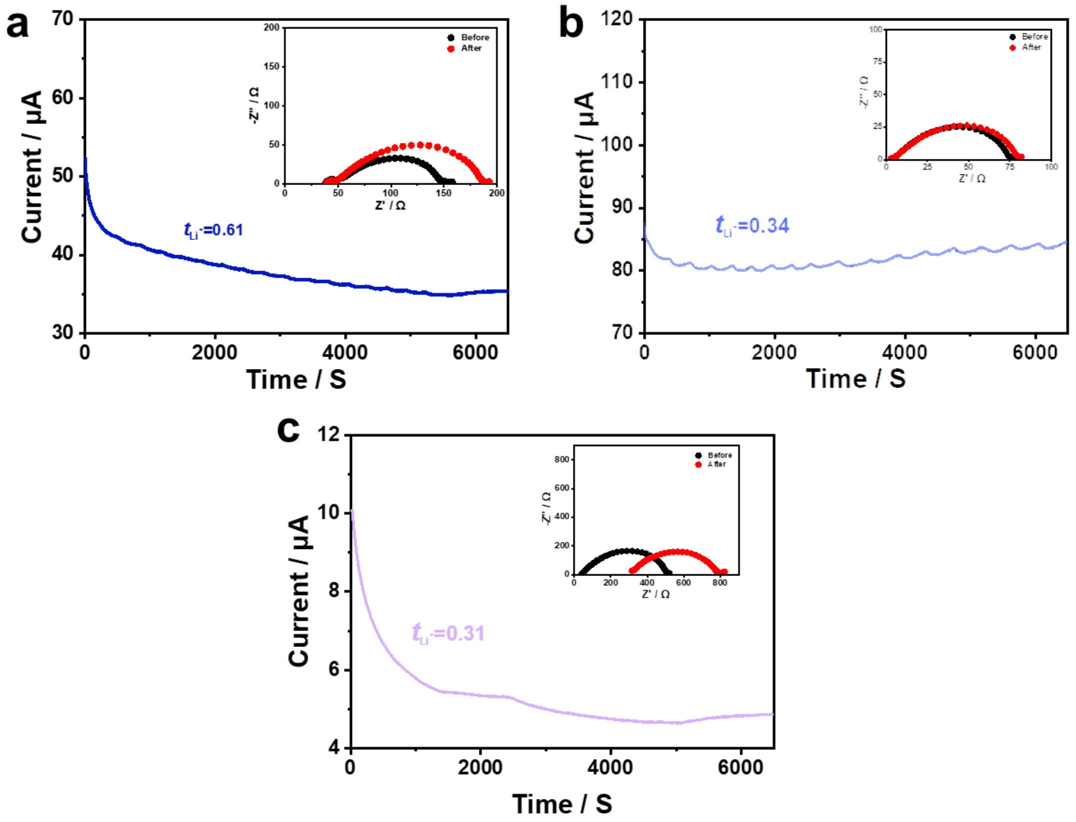

Supplementary Fig. 15. Current-time profiles of Li/PVFH-PVCA/Li (**a**), Li/CLE/Li (**b**) and Li/PVLE/Li (**c**) symmetric batteries applied by a DC voltage of 10 mV. The inset shows the EIS of the battery before and after polarization.

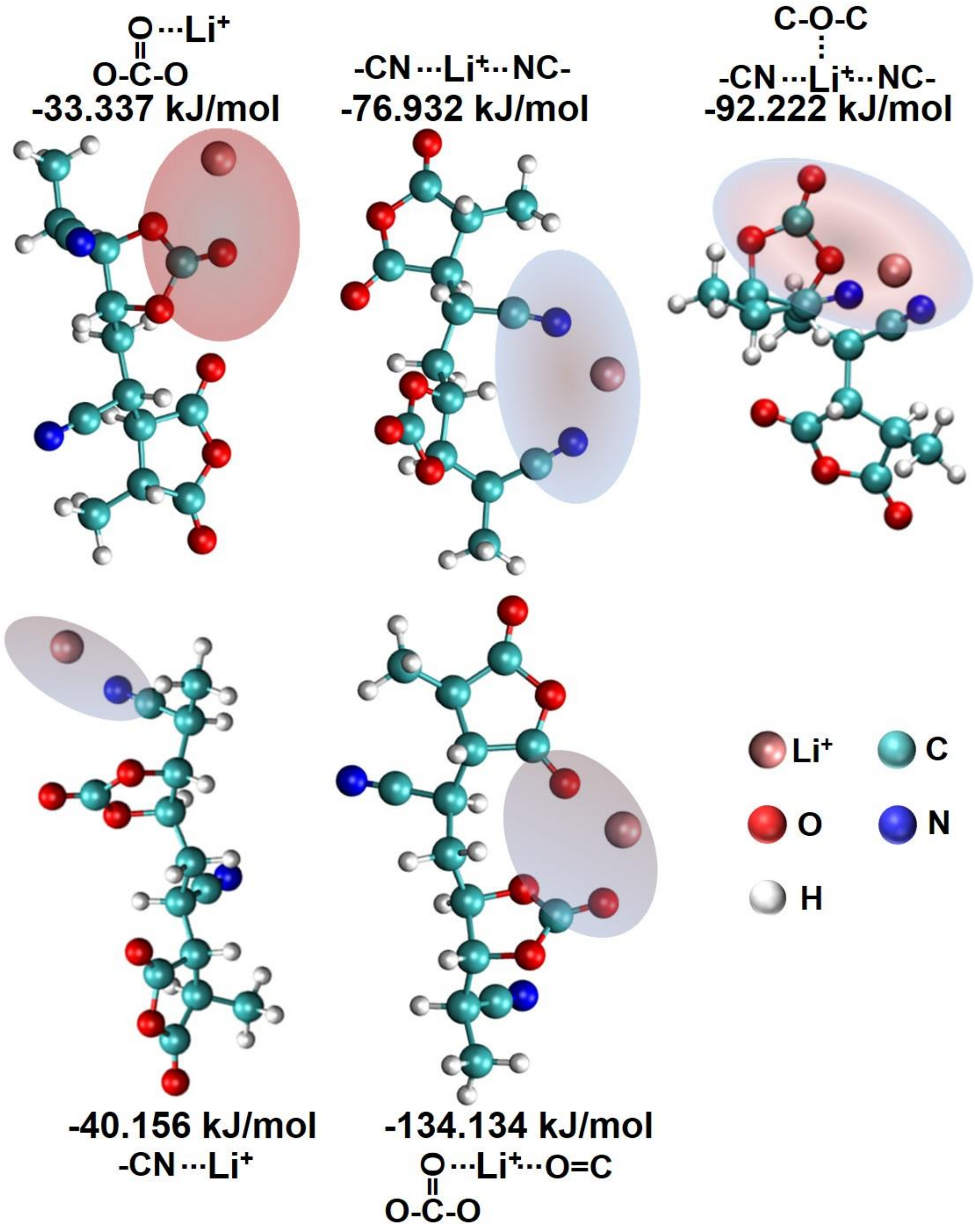

Supplementary Fig. 16. The PVCA molecule–ion interactions. Three polar groups are demonstrated to synergistically improve Li^+ movement (higher Li^+ conductivity and higher Li^+ transfer number).

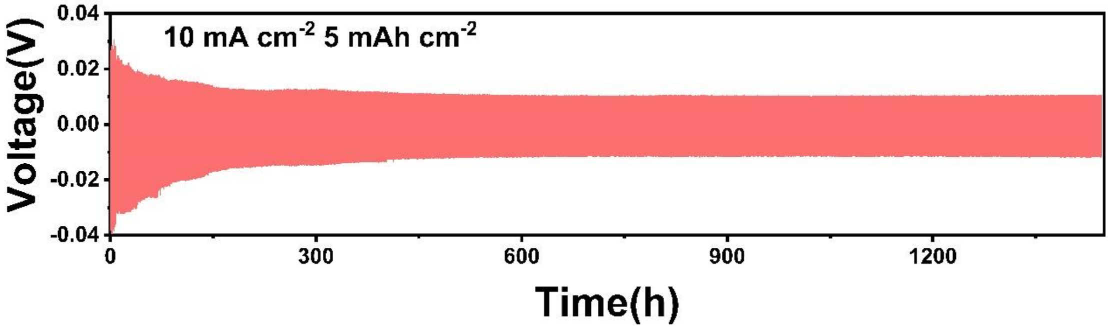

Supplementary Fig. 17. Galvanostatic cycling curves of Li/Li symmetric batteries using PVFH-PVCA electrolytes at 10 mA cm^{-2} and 5 mAh cm^{-2}.

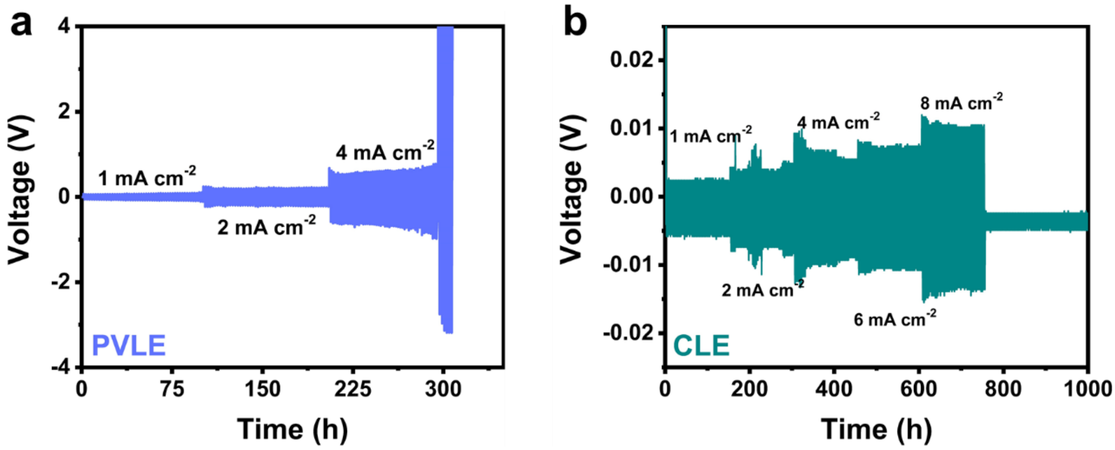

Supplementary Fig. 18. Voltage versus time curves at current densities from 1 to 8 mA cm^{-2}. (**a**). Li/Li symmetric batteries using PVLE electrolytes. (**b**) Li/Li symmetric batteries using CLE.

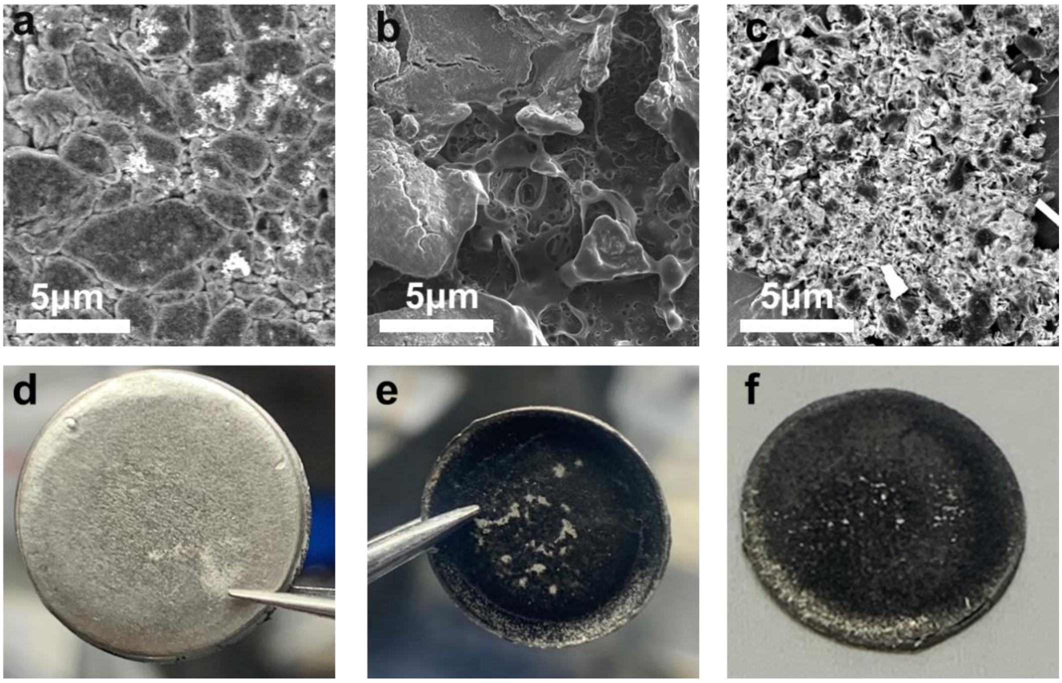

Supplementary Fig. 19. SEM images (**a-c**) and Li anode digital pictures(**d-f**). Symmetrical Li||Li cells utilizing PVFH-PVCA (**a** and **d**), CLE (**b** and **e**) and PVLE (**c** and **f**) after the 20 cycles.

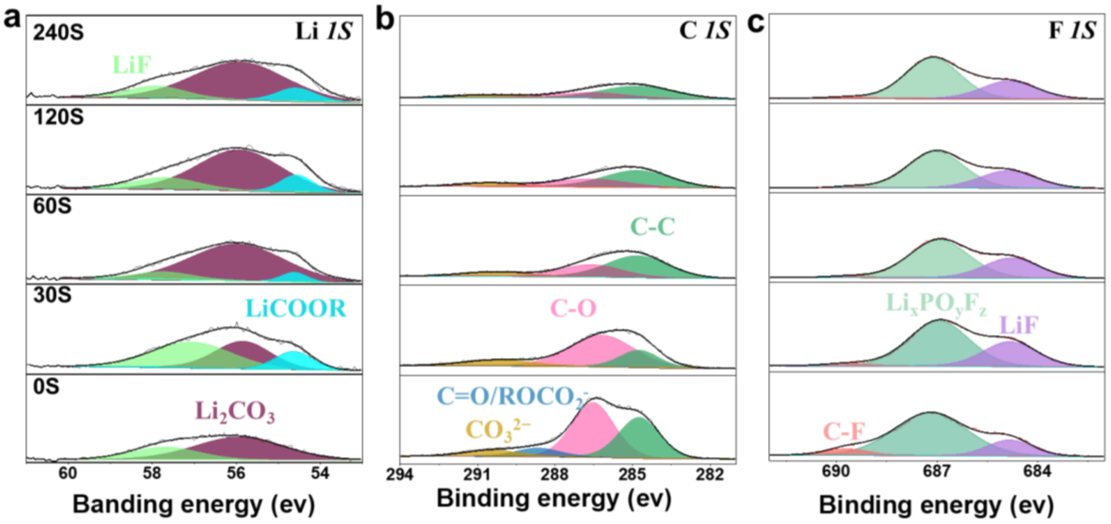

Supplementary Fig. 20. Li *1s*, C *1s* and F *1s* in-depth XPS spectra of Li metal anodes from Li symmetric cells using CLE electrolyte after cycles.

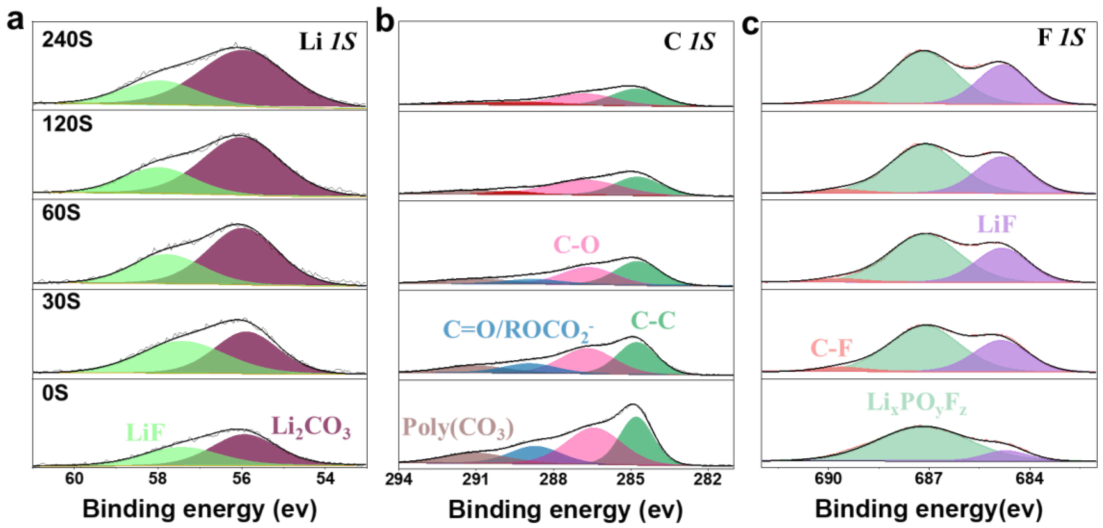

Supplementary Fig. 21. Li *1s*, C *1s* and F *1s* in-depth XPS spectra of Li metal anodes from Li symmetric cells using PVLE electrolyte after cycles.

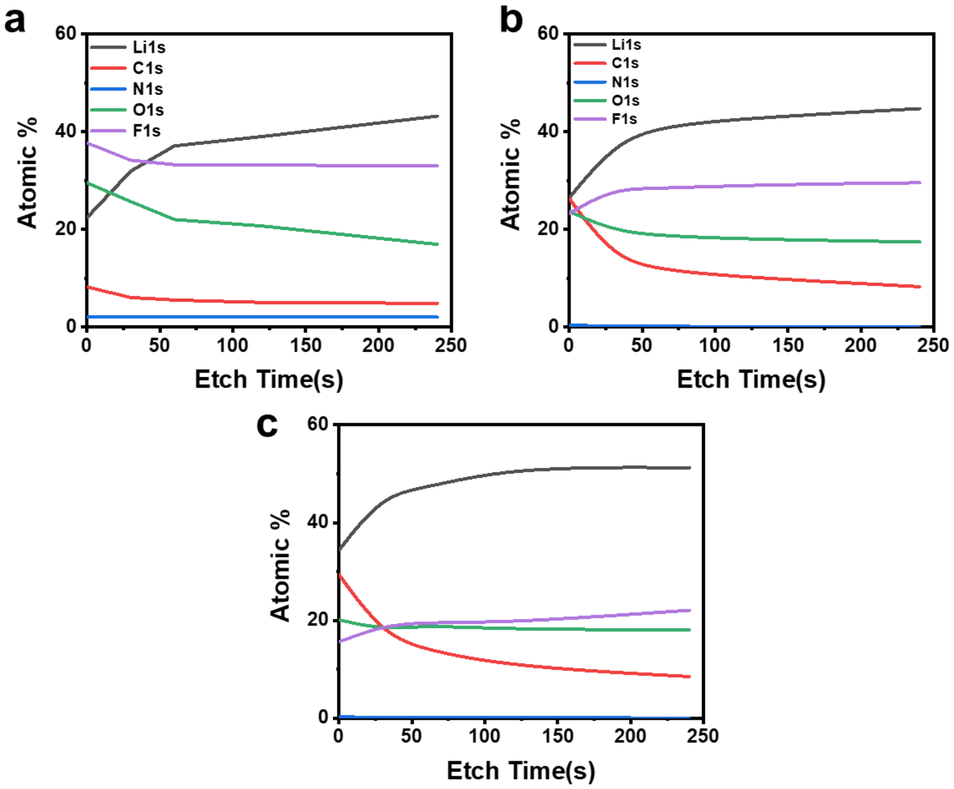

Supplementary Fig. 22. Elemental analyses of the cycled Li electrodes collected from symmetrical Li||Li cells utilizing PVFH-PVCA (**a**), CLE(**b**) and PVLE (**c**) after the 20 cycles.

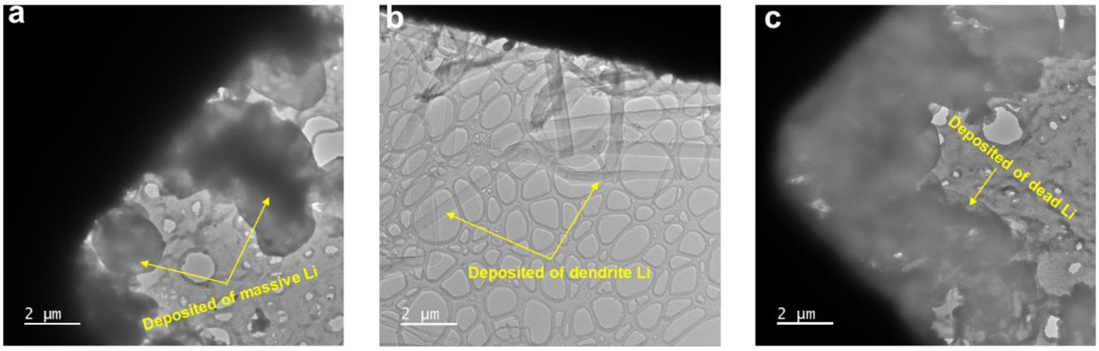

Supplementary Fig. 23. Morphology of lithium deposited on Cu grid. a-c, Cryo-TEM images of the deposited Li after the first stripping using PVFH-PVCA(**a**), CLE(**b**) and PVLE(**c**).

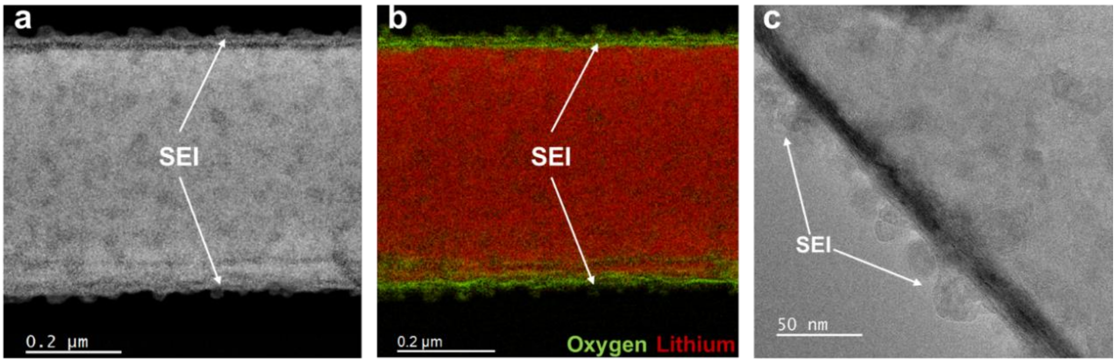

Supplementary Fig. 24. Representative cryo-STEM ADF image (**a**) and EELS map (**b**) of deposited Li using CLE. (**c**) Uneven SEI layer on the surface of lithium dendrites deposited.

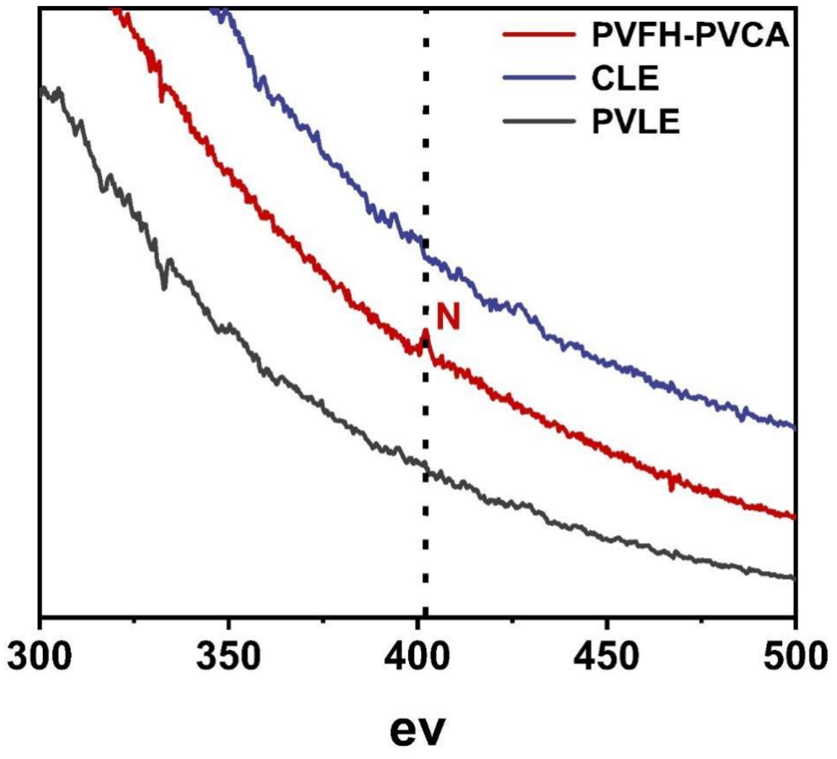

Supplementary Fig. 25. EELS Spectrum of SEI formed by different electrolyte cycles. The SEI formed by PVFH-PVCA electrolyte cycle is rich in N element, and the characteristic peak of ~404 ev is N.

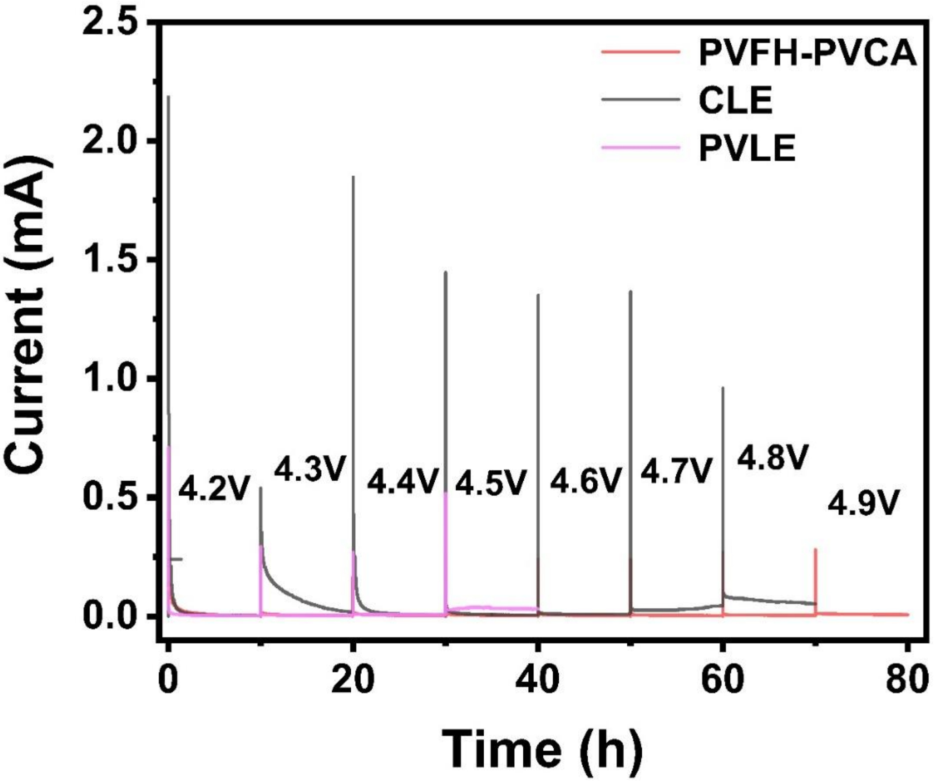

Supplementary Fig. 26. Electrochemical floating experiment was performed using Li|| LMNCO with PVFH-PVCA, CLE and PVFH, respectively. The cell was charged to 4.2 V at 0.2C and then held at gradually higher voltages for 10 h up to 5.0 V. The results reported show that at voltages below 4.9 V, the leakage current measured in PVFH-PVCA is small (< 20 μA). In contrast, similar experiments using the CLE and PVLE reveal leakage currents exceeding 96 μA and 40μA at voltages as low as 4.8 V and 4.5 V, respectively.

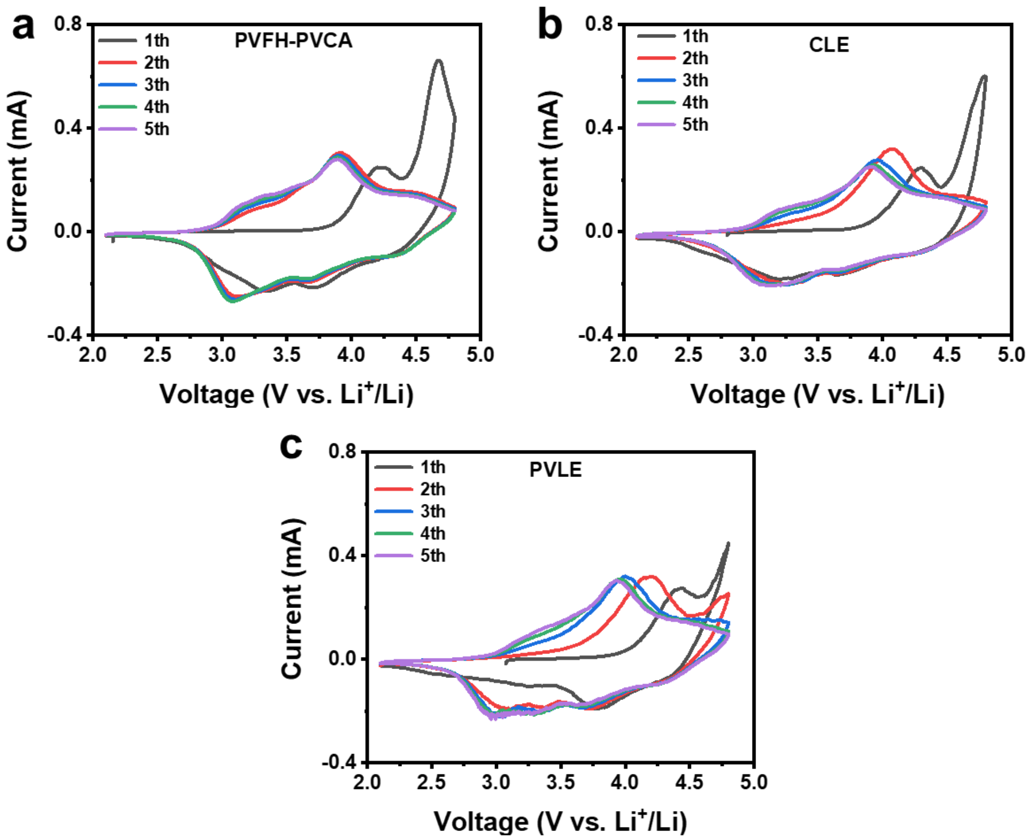

Supplementary Fig. 27. Cyclic voltammetry curves of (**a**) Li| PVFH-PVCA |LMNCO cells, (**b**) Li| CLE | LMNCO cells and (**c**) Li| PVLE | LMNCO cells, 25 °C between 2.1 and 4.8 V. Sweep rate: 1mV s^{-1}.

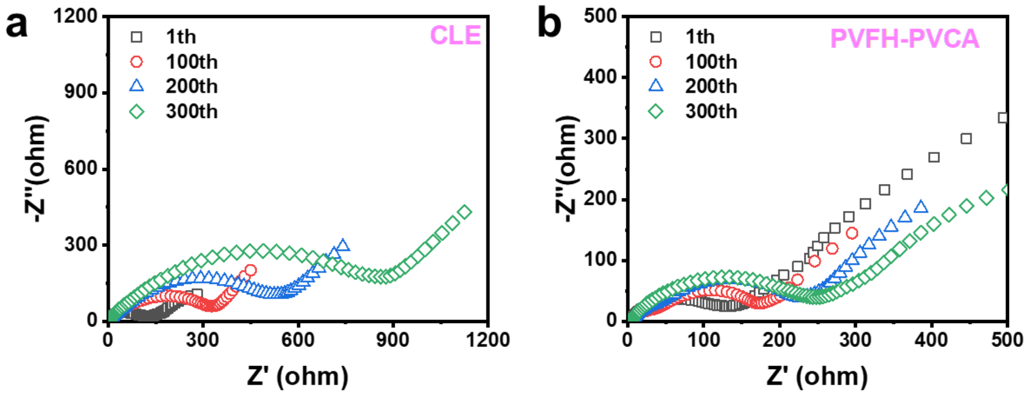

Supplementary Fig. 28. Nyquist plots of Li // LMNCO batteries at selected cycles in (**a**) CLE and (**b**) PVFH-PVCA.

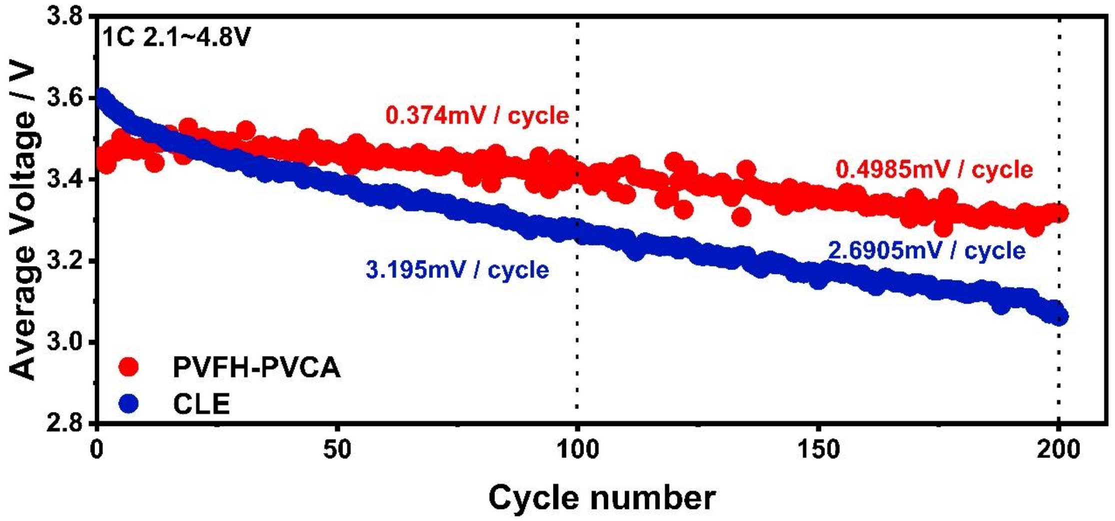

Supplementary Fig. 29. The average discharge voltage of the Li-rich cathode using PVFH-PVCA electrolyte and CLE electrolyte upon increasing cycle numbers. When cycled at a current density of 1 C in the voltage window of 2.1–4.8 V, the voltage retention of the PVFH-PVCA is as high as 97.1% after 200 cycles. The voltage degradation per cycle is less than 0.05%, with a voltage loss of ≈0.5 mV per cycle, which is excellent performance. However, the voltage loss in the CLE reaches 538.1 mV after 200 cycles.

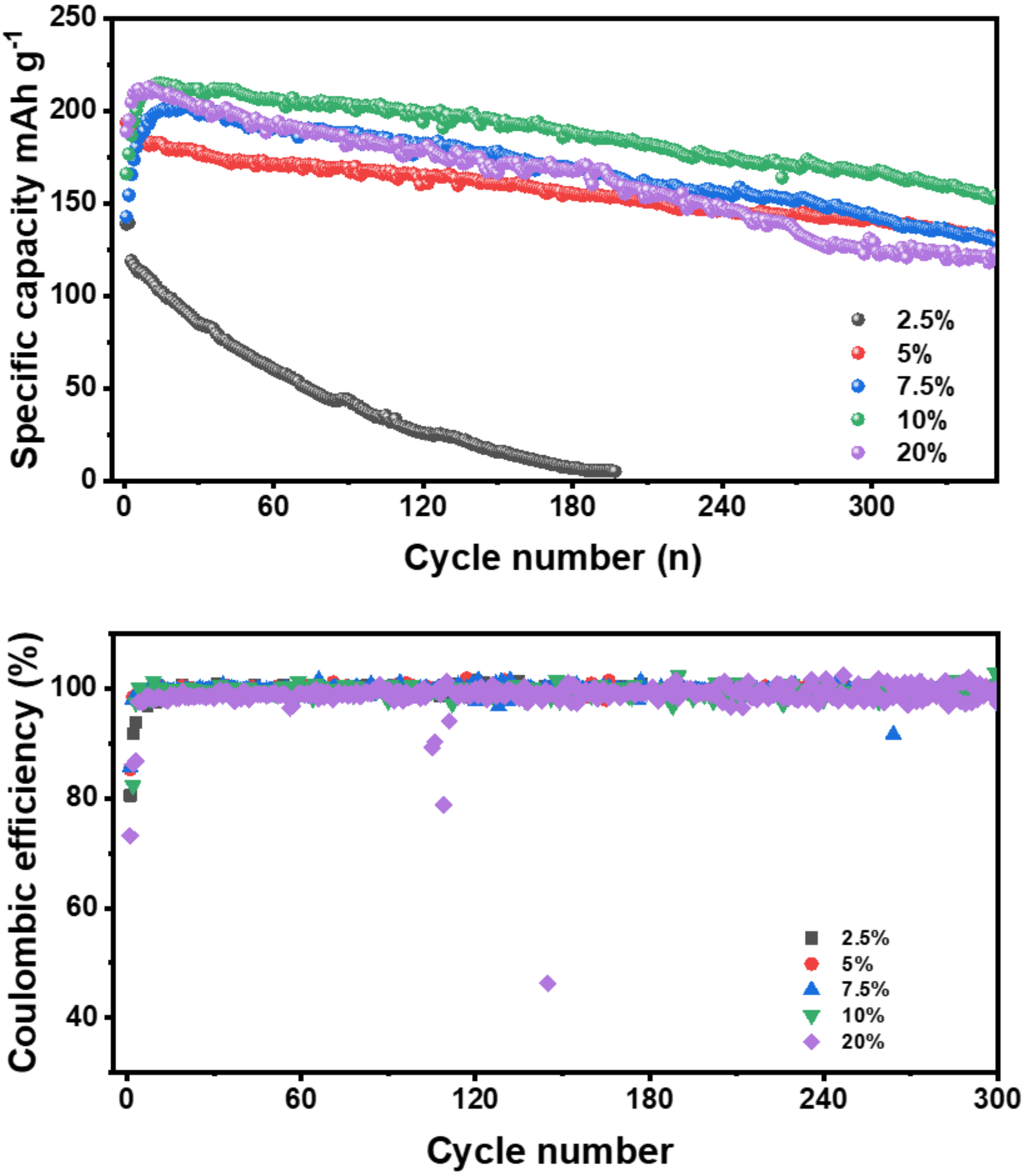

Supplementary Fig. 30. Cycling performance of Li||LMNCO cells with different content of PVCA at 1 C at 30 °C between 2.1 V and 4.6 V.

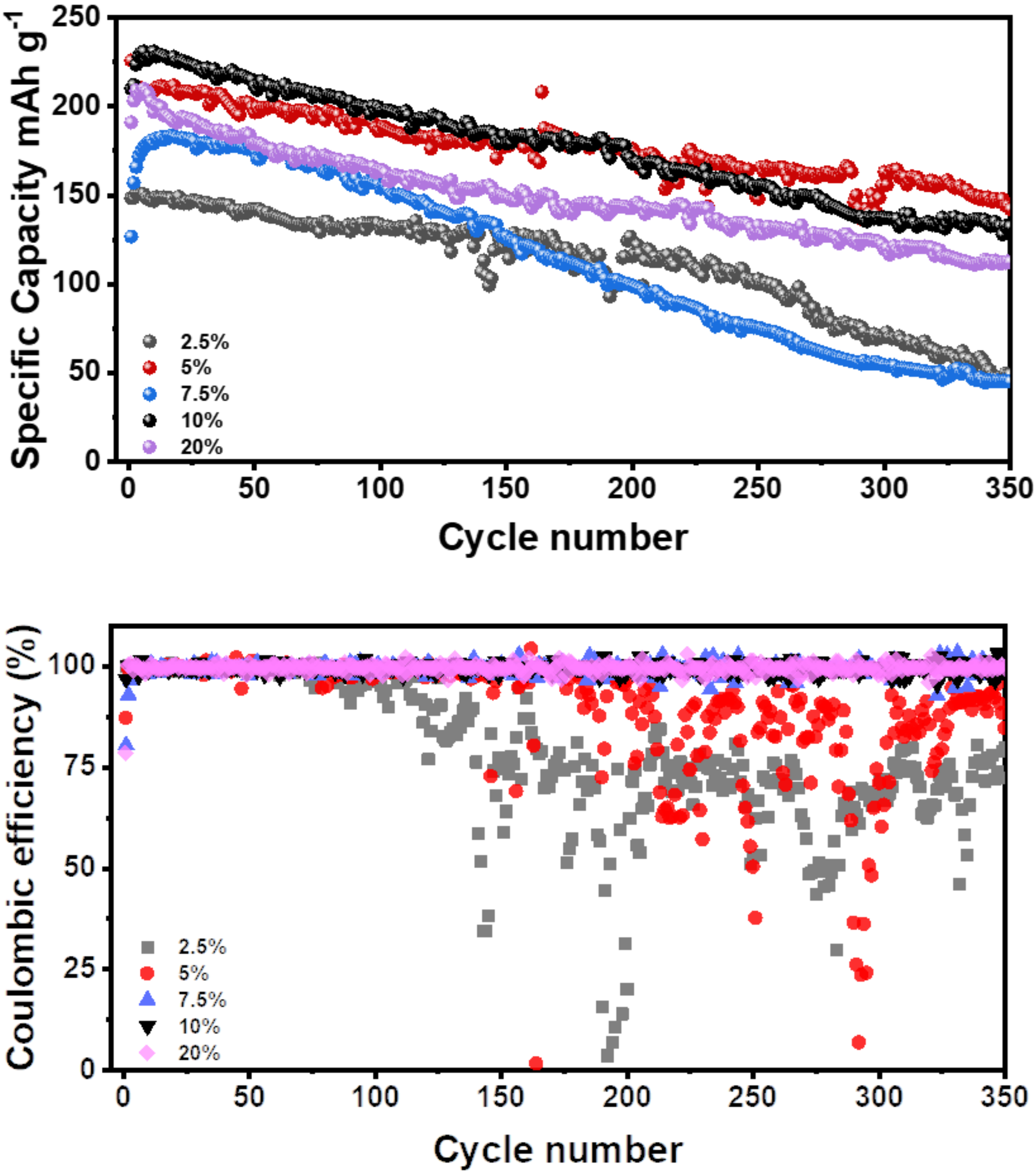

Supplementary Fig. 31. Cycling performance of Li||LMNCO cells with different content of PVCA at 1 C at 30 °C between 2.1 V and 4.8 V.

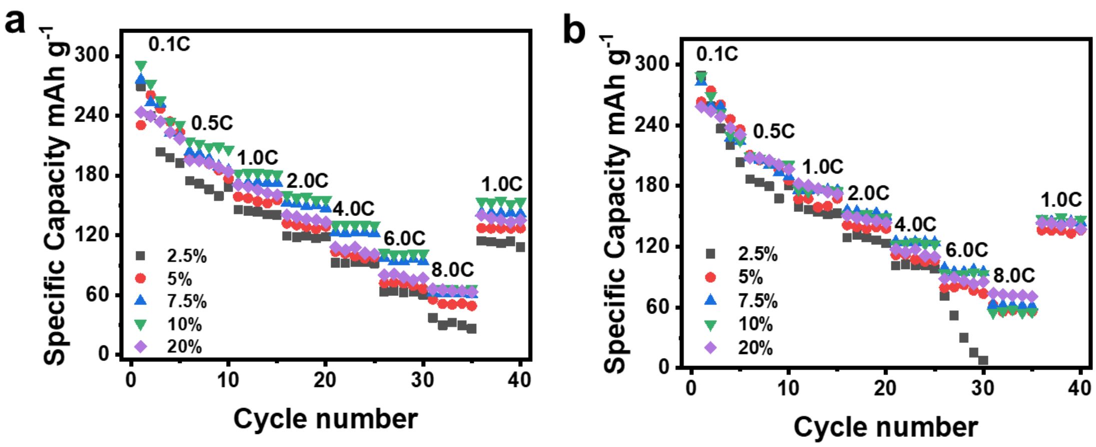

Supplementary Fig. 32. Rate performance of Li||LMNCO cells with different content of PVCA at 30 °C between 2.1 V and 4.6 V(a) and between 2.1V and 4.8V(b).

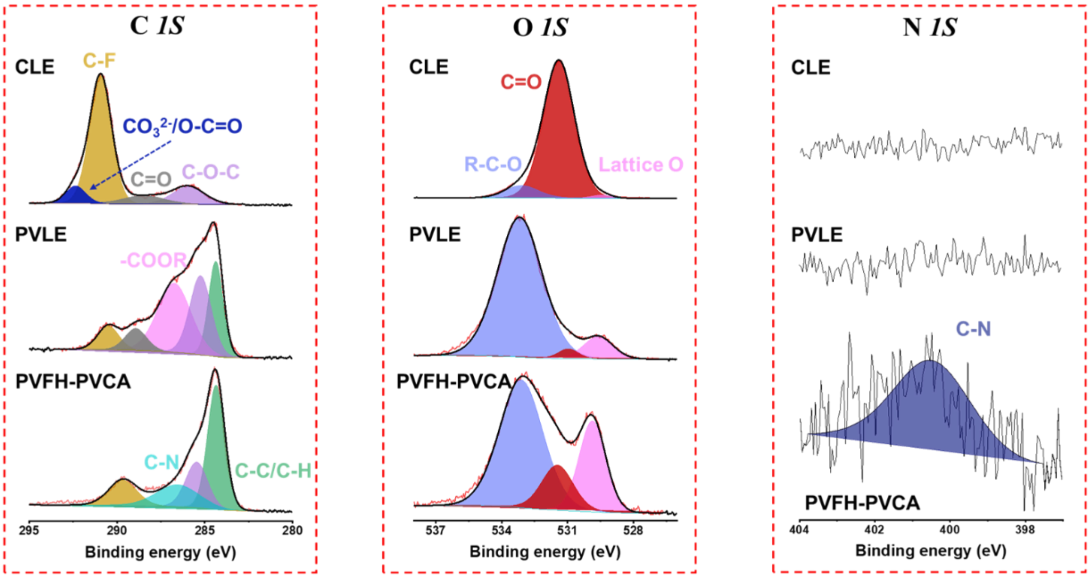

Supplementary Fig. 33|. Characterization of the CEI components on the cycled LMNCO cathodes with CLE, PVLE and PVFH-PVCA by XPS. The high-resolution C 1s, O 1s and N 1s XPS spectra of the LMNCO cathodes were measured after 50 cycles of the Li//LMNCO cells with CLE, PVLE and PVFH-PVCA at 1C and 2.1~4.8V.

Supplementary Tab. 1. Electrochemical and mechanical performance of Li batteries with gel polymer electrolytes reported in the literature.

Electrolytes	IC ($mS\cdot cm^{-1}$)	ESW (V)	Stress (MPa)	BC	LC ($mAh\ g^{-1}$)	C-rate	Cycle	CR (%)	References
PVDF/DMIm/LiTFSI	1.67	4.35	1.0	Li||LFP	150.7	0.1	100	99.1	1
PEGGE/$LiPF_6$	0.27	5.0	5.5	Li||LFP	146.8	0.2	360	88.49	2
PVDF-HFP/MXene/EmimTFSI	1.54	4.7	~9.0	Li||LFP	151	0.2	200	97.8	3
PVDF-HFP/ZIF8/Al_2O_3	1.67	5.0	13.08	Li|| LNCMO	257.5	0.2	500	84.6	4
PVDF/[Li(DMF)$_n$][TFSI]	1.55	4.97	3.4	Li||NCM622	142.7	0.1	500	96	5
PVDF/organo-polysulfide polymer	0.708	4.5	10.83	Li||S	-	0.5	300	57.4	6
PEDG/TAC/$LiPF_6$	1.08	5.0	0.7	Li||LFP	155.2	0.2	200	90.5	7
PEGDE/DPPO/DEBA	2.36	4.2	7.6	Li||LFP	154.3	0.3	200	93.2	8
PVDF-HFP/$Li_{6.4}Ga_{0.2}La_3Zr_2O_{12}$	1.84	4.75	~7.1	Li||NCM523	128.6	0.5	360	94.08	9
CNF/PEGDME/Celluiose /LiTFSI	0.023	4.0	9.5	Li||LFP	~125	1.0(60°C)	300	94	10
PVFH-PVCA	2.04	5.3	19.83	Li||LFP	154.9	0.5	1940	86.4	This work
				Li||LCO	167.7	1.0	1500	89.2	
				Li||NC95	207.1	1.0	200	78.7	
				Li|| LMNCO	214.1 (4.6V)	1.0	400	84.8	
					238.4 (4.8V)		340	78.8	

Nomenclature: IC: Ionic conductivity; ESW: Electrochemical stability window; BC: battery configuration; LC: Initial capacity; CR: Capacity retention; LMNCO: $Li_{1.2}Mn_{0.56}Ni_{0.16}Co_{0.08}O_2$; LNCMO: $Li_{1.2}Ni_{0.13}Co_{0.13}Mn_{0.54}O_2$

Supplementary References

1 Pei, X. *et al.* Li−N Interaction Induced Deep Eutectic Gel Polymer Electrolyte for High Performance Lithium-Metal Batteries. *Angewandte Chemie International Edition* **61**, e202205075, doi:https://doi.org/10.1002/anie.202205075 (2022).

2 Xu, D., Jin, J., Chen, C. & Wen, Z. From Nature to Energy Storage: A Novel Sustainable 3D Cross-Linked Chitosan–PEGGE-Based Gel Polymer Electrolyte with Excellent Lithium-Ion Transport Properties for Lithium Batteries. *ACS Applied Materials & Interfaces* **10**, 38526-38537, doi:10.1021/acsami.8b15247 (2018).

3 Tang, Y. *et al.* A Solid-State Lithium Battery with PVDF–HFP-Modified Fireproof Ionogel Polymer Electrolyte. *ACS Applied Energy Materials* **6**, 4016-4026, doi:10.1021/acsaem.3c00249 (2023).

4 Cui, S. *et al.* Heterostructured Gel Polymer Electrolyte Enabling Long-Cycle Quasi-Solid-State Lithium Metal Batteries. *ACS Energy Letters* **7**, 42-52, doi:10.1021/acsenergylett.1c02233 (2022).

5 Xu, F., Deng, S., Guo, Q., Zhou, D. & Yao, X. Quasi-Ionic Liquid Enabling Single-Phase Poly(vinylidene fluoride)-Based Polymer Electrolytes for Solid-State LiNi0.6Co0.2Mn0.2O2||Li Batteries with Rigid-Flexible Coupling Interphase. *Small Methods* **5**, 2100262, doi:https://doi.org/10.1002/smtd.202100262 (2021).

6 Shen, Y.-Q. *et al.* A novel permselective organo-polysulfides/PVDF gel polymer electrolyte enables stable lithium anode for lithium–sulfur batteries. *Journal of Energy Chemistry* **48**, 267-276, doi:https://doi.org/10.1016/j.jechem.2020.01.016 (2020).

7 Liao, H., Chen, H., Zhou, F., Zhang, Z. & Chen, H. Dendrite-free lithium deposition induced by mechanical strong sponge-supported unique 3D cross-linking polymer electrolyte for lithium metal batteries. *Journal of Power Sources* **435**, 226748, doi:https://doi.org/10.1016/j.jpowsour.2019.226748 (2019).

8 Lu, Q. *et al.* Dendrite-Free, High-Rate, Long-Life Lithium Metal Batteries with a 3D Cross-Linked Network Polymer Electrolyte. *Advanced Materials* **29**, 1604460, doi:https://doi.org/10.1002/adma.201604460 (2017).

9 Xu, D. *et al.* In Situ Generated Fireproof Gel Polymer Electrolyte with Li6.4Ga0.2La3Zr2O12 As Initiator and Ion-Conductive Filler. *Advanced Energy Materials* **9**, 1900611, doi:https://doi.org/10.1002/aenm.201900611 (2019).

10 Wang, Z. *et al.* Dynamic Networks of Cellulose Nanofibrils Enable Highly Conductive and Strong Polymer Gel Electrolytes for Lithium-Ion Batteries. *Advanced Functional Materials* **n/a**, 2212806, doi:https://doi.org/10.1002/adfm.202212806.
